# Comparison of semi-automated methods to quantify infarct size and area at risk by cardiovascular magnetic resonance imaging at 1.5T and 3.0T field strengths

**DOI:** 10.1186/s13104-015-1007-1

**Published:** 2015-02-25

**Authors:** Jamal N Khan, Sheraz A Nazir, Mark A Horsfield, Anvesha Singh, Prathap Kanagala, John P Greenwood, Anthony H Gershlick, Gerry P McCann

**Affiliations:** Department of Cardiovascular Sciences, University of Leicester and the NIHR Leicester Cardiovascular Biomedical Research Unit, Glenfield Hospital, Groby Road, LE3 9QP Leicester, UK; Division of Cardiovascular and Diabetes Research, Leeds Institute of Genetics, Health and Therapeutics, University of Leeds, LS2 9JT Leeds, UK

**Keywords:** Myocardial infarction, Late gadolinium enhancement, Infarct size, T2-weighted STIR, Myocardial salvage, Myocardial oedema, Ischaemic area at risk

## Abstract

**Background:**

There is currently no gold standard technique for quantifying infarct size (IS) and ischaemic area-at-risk (AAR [oedema]) on late gadolinium enhancement imaging (LGE) and T2-weighted short tau inversion recovery imaging (T2w-STIR) respectively. This study aimed to compare the accuracy and reproducibility of IS and AAR quantification on LGE and T2w-STIR imaging using Otsu’s Automated Technique (OAT) with currently used methods at 1.5T and 3.0T post acute ST-segment elevation myocardial infarction (STEMI).

**Methods:**

Ten patients were assessed at 1.5T and 10 at 3.0T. IS was assessed on LGE using 5–8 standard-deviation thresholding (5-8SD), full-width half-maximum (FWHM) quantification and OAT. AAR was assessed on T2w-STIR using 2SD and OAT. Accuracy was assessed by comparison with manual quantification. Interobserver and intraobserver variabilities were assessed using Intraclass Correlation Coefficients and Bland-Altman analysis. IS using each technique was correlated with left ventricular ejection fraction (LVEF).

**Results:**

FWHM and 8SD-derived IS closely correlated with manual assessment at both field strengths (1.5T: 18.3 ± 10.7% LV Mass [LVM] with FWHM, 17.7 ± 14.4% LVM with 8SD, 16.5 ± 10.3% LVM with manual quantification; 3.0T: 10.8 ± 8.2% LVM with FWHM, 11.4 ± 9.0% LVM with 8SD, 11.5 ± 9.0% LVM with manual quantification). 5SD and OAT overestimated IS at both field strengths. OAT, 2SD and manually quantified AAR closely correlated at 1.5T, but OAT overestimated AAR compared with manual assessment at 3.0T. IS and AAR derived by FWHM and OAT respectively had better reproducibility compared with manual and SD-based quantification. FWHM IS correlated strongest with LVEF.

**Conclusions:**

FWHM quantification of IS is accurate, reproducible and correlates strongly with LVEF, whereas 5SD and OAT overestimate IS. OAT accurately assesses AAR at 1.5T and with excellent reproducibility. OAT overestimated AAR at 3.0T and thus cannot be recommended as the preferred method for AAR quantification at 3.0T.

**Electronic supplementary material:**

The online version of this article (doi:10.1186/s13104-015-1007-1) contains supplementary material, which is available to authorized users.

## Background

Cardiovascular magnetic resonance (CMR)-measured infarct size (IS) [1,2] and myocardial salvage index (MSI) [3,4] are important measures of reperfusion success and predictors of remodelling and prognosis post acute ST-segment elevation myocardial infarction (STEMI). MSI is the proportion of reversibly injured ischaemic area-at-risk (AAR) visualised as myocardium with high signal intensity on T2-weighted images [3-5].

There is currently no gold standard technique for the quantification of IS and AAR on late gadolinium imaging (LGE) and T2-weighted short tau inversion recovery imaging (T2w-STIR) respectively.[6] Semi-automated standard deviation (SD)-based thresholding techniques [4,7], manual (visual) contouring of enhancement [1,2], the full-width half-maximum (FWHM) method [6,8], and recently, automated techniques have been used [9,10]. The heterogeneity of techniques and resulting IS and AAR values hinders comparisons between studies.

Otsu’s Automated Thresholding (OAT) automatically identifies hyperenhanced areas by selecting the grayscale signal intensity threshold giving minimal intraclass variance within enhanced and normal myocardium and is largely user-independent [11]. There are very scarce published data using OAT quantification, of IS [12] and AAR [13,14].

There are no published studies assessing IS or AAR quantification at 3.0T, or using 7SD and 8SD infarct quantification thresholding at any field strength.

This study aimed to compare the accuracy and reproducibility of IS and AAR quantification on LGE and T2w-STIR using OAT with the currently used quantification methods at 1.5T and 3.0T.

## Methods

### Study population

Ten patients were retrospectively, randomly selected using a random number generator [15] from the cohort of a UK multicentre, prospective CMR study investigating acute STEMI management at 1.5T (Complete Versus culprit-Lesion only PRimary PCI Trial) [16]. Ten further patients were identically selected from a separate multicentre study at 3.0T (Randomized Controlled Trial Comparing Intracoronary Administration of Adenosine or Sodium Nitroprusside to Control for Attenuation of Microvascular Obstruction During Primary Percutaneous Coronary Intervention) [17]. STEMI was diagnosed according to ESC definitions [18] and patients underwent primary PCI within 12 h of symptom onset. The studies were approved by Trent Research Ethics Committee, conducted according to the Declaration of Helsinki and all participants provided written informed consent.

### CMR image acquisition

**Figure 1:**
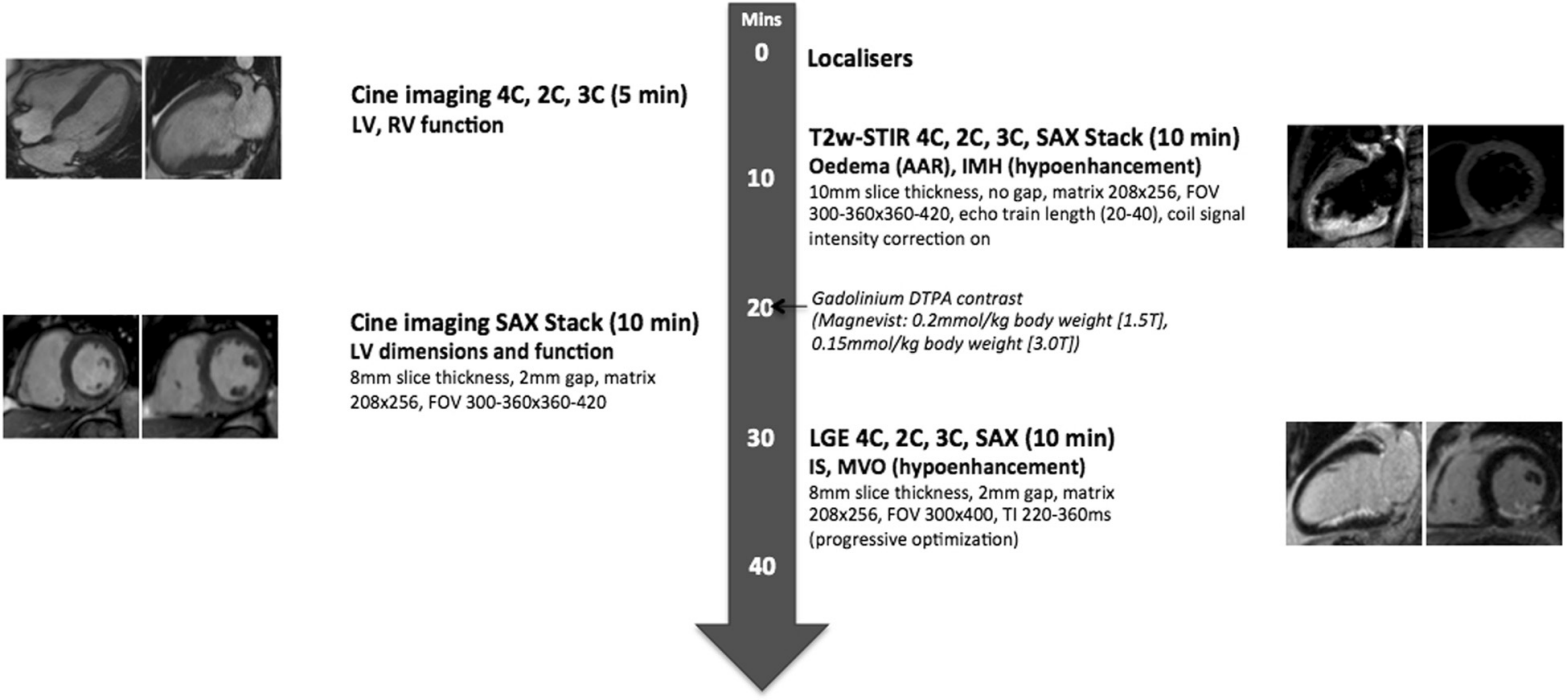
**CMR protocol (for Siemens Avanto 1.5T scanner; parameters for other scanners presented in Additional file** 1 **).** 4C, 2C, 3C = 4,2,3-chamber long-axis views, LV = left ventricle, SAX = short-axis, FOV = field of view, AAR = area at risk, IS = infarct size, IMH = intramyocardial haemorrhage, MVO = microvascular obstruction.

CMR was performed during the index admission on a 1.5T scanner (Siemens Avanto, Erlangen, Germany [n = 4] or Philips Intera, Best, The Netherlands [n = 6]) or 3.0T scanner (Siemens Skyra, Erlangen, Germany [n = 5]; Philips Achieva TX, Best, Netherlands [n = 4] or GE Signa HDxt, Little Chalford, UK [n = 1]) with retrospective electrocardiographic gating and dedicated cardiac receiver coils. The imaging protocol is outlined in Figure 1 and Additional file 1. T2w-STIR imaging with coil SI correction, cine imaging with steady state free precession and Late Gadolinium Enhancement (LGE) imaging were performed in long-axis views and contiguous short-axis slices covering the entire LV. LGE images were acquired 10–15 minutes after administration of 0.15 mmol/kg (3.0T) or 0.2 mmol/kg (1.5T) gadolinium-DTPA (Magnevist, Bayer, Germany) using a segmented inversion-recovery gradient-echo sequence. The inversion time was progressively adjusted to null unaffected myocardium.

### IS and AAR quantification

Image quality was graded according to a 4-point scale before analysis: 3 = excellent, 2 = good, 1 = moderate and 0 = unanalysable. To remove the confounding variable of image quality on AAR quantification, 26% of studies from the total study population, where T2w-STIR images were deemed non-analysable were excluded from the random number study selection pool. Analysis was performed offline in a central core lab, blinded to patient details using *cmr42* (Circle Cardiovascular Imaging, Calgary, Canada). LGE, T2w-STIR and cine images were studied together and co-registered to allow accurate quantification based on all available data. For the assessment of LV volumes and function, IS and AAR, endocardial and epicardial borders were manually contoured on contiguous short-axis LV slices, excluding papillary muscles, trabeculae, epicardial surfaces and blood-pool artefact, and the quantification method applied. The most apical LGE and T2w-STIR slice was excluded to minimize partial volume effect. Total IS and AAR were expressed as percentage of LV mass (LVM).

#### IS quantification

**Figure 2:**
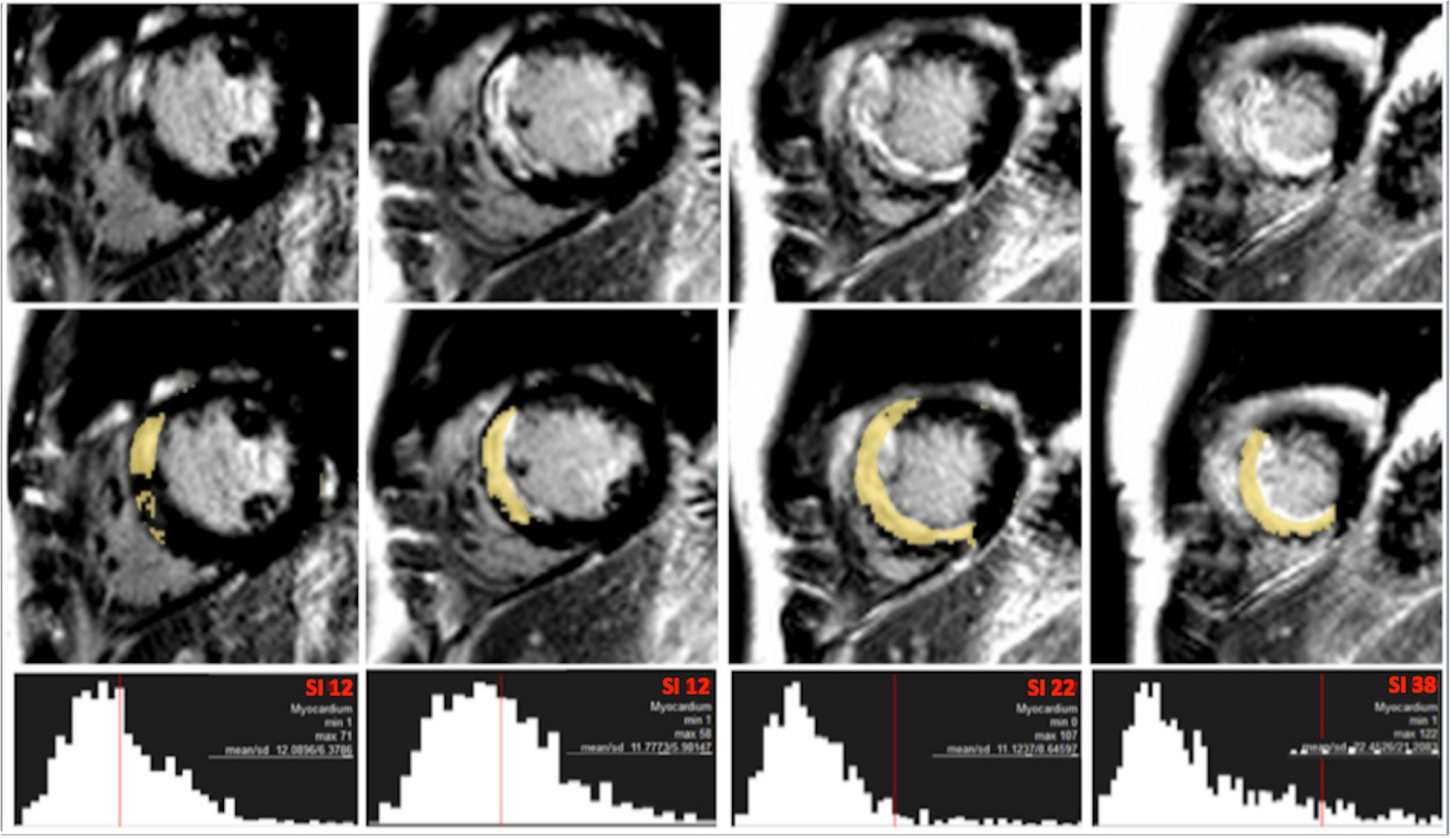
**Otsu’s Automated Thresholding (OAT) method.** *Top row: Short-axis late gadolinium images from basal to apical (left to right). Middle row: Enhancement (yellow) signifying infarct, designated on a slice-by-slice basis by OAT method. Bottom row: OAT automatically identifies hyperenhanced areas by selecting the grayscale signal intensity threshold (red) on a slice-by-slice basis that gives the minimal intraclass variance within enhanced and normal myocardium.*

IS was quantified on LGE magnitude images as hyperenhancement using 5/6/7/8 SD thresholding, FWHM [8] and OAT by 2 experienced readers (JNK, SN: 3 years experience each). Mean IS was compared using the techniques and with manual (visual) quantification. As there is no gold standard technique for *in vivo IS quantification, we used* the mean of 6 analyses (manual quantification undertaken twice each by observers JNK and SAN, and by an SCMR Level 3 trained reader [GPM: 10 years experience]). Manual quantification has been used in this capacity in the majority of studies comparing quantification methods for IS [6,19,20] and AAR [13,21,22], and has high intraobserver and interobserver agreement and reproducibility [6,14]. For 5/6/7/8 SD thresholding, a region of interest (ROI) was manually drawn in remote (no enhancement, oedema or wall-motion abnormality) myocardium and the area of enhancement automatically calculated as the region with signal intensity 5/6/7/8 SD above the mean within the ROI respectively. For the FWHM technique, an ROI was manually drawn in the infarct core and enhancement calculated as pixels where signal intensity exceeded 50% of the automatically determined maximum signal intensity in the infarct core. Where it was not obvious which ROI in the infarct core had the highest maximum signal intensity, ROIs were drawn in potential regions and the ROI with the highest signal intensity selected. The ROI size for the 5/6/7/8 SD and FWHM methods was set at 2 cm^2^. The FWHM method is unaffected by ROI size as it selects the threshold based on the single pixel with highest signal intensity. The same signal intensity threshold was set for all slices on 5/6/7/8 SD and FWHM thresholding. OAT automatically calculates a unique signal intensity threshold for each slice by dividing the greyscale signal intensity histogram in each slice into 2 groups (enhanced, normal) based on the signal intensity threshold giving the least intraclass variance (lowest sum of variances) and thus most homogeneity of signal intensities within each group (Figure 2) [11,12]. The only user input, and thus potential sources of variation are the endocardial and epicardial contours, and manual correction of noise artefact. OAT requires no ROI selection and is thus largely user-independent compared with SD-based, FWHM and manual quantification.

#### AAR quantification

AAR was quantified on T2w-STIR as hyperenhancement using 2SD thresholding and OAT by 2 blinded readers (JNK, SAN). The ROI size for 2SD was set at 2 cm^2^. Mean AAR was compared across the techniques and with manual quantification as described above for IS quantification.

Two manual corrections were applied to IS and AAR measurements: [a] inclusion of hypointense regions within enhancement corresponding to microvascular obstruction and intramyocardial haemorrhage in total IS and AAR respectively [4,6]; [b] exclusion of small isolated regions of enhancement without interslice continuity, in non-infarct related artery territories deemed to be noise artefact.

To assess intraobserver variability of the techniques, all images were re-quantified by a single observer after a 2-month interval. We also recorded the time taken to quantify IS and AAR using each of the methods once the endocardial and epicardial contours had been drawn (time taken for [a] quantification of AAR or IS using chosen technique + [b] inclusion of IMH or MVO where appropriate + [c] exclusion of noise artefact).

### Statistical analysis

Normality was assessed using the Shapiro-Wilk test, histograms and Q-Q plots. Normally distributed data were expressed as mean ± standard deviation. IS and AAR by each quantification method were normally distributed and thus compared using paired t-tests, and the accuracy of each method was assessed by comparison with manual assessment using paired t-testing, two-way mixed-effect intraclass correlation coefficient for absolute agreement ICC (three, one) [23] and Bland-Altman analysis [24]. On ICC, agreement was defined as excellent (ICC ≥ 0.75), good (ICC 0.6-0.74), fair (ICC 0.4-0.59), or poor (ICC < 0.40) [25]. Interobserver and intraobserver variabilities were expressed using ICC (three, one) and Bland-Altman analysis. The significance of differences in reproducibility was assessed using Wilcoxon rank comparison of the squared differences [6]. Statistical tests were performed using SPSS v20 (IBM, USA). p < 0.05 was considered significant.

## Results

### Baseline characteristics

**Figure 3:**
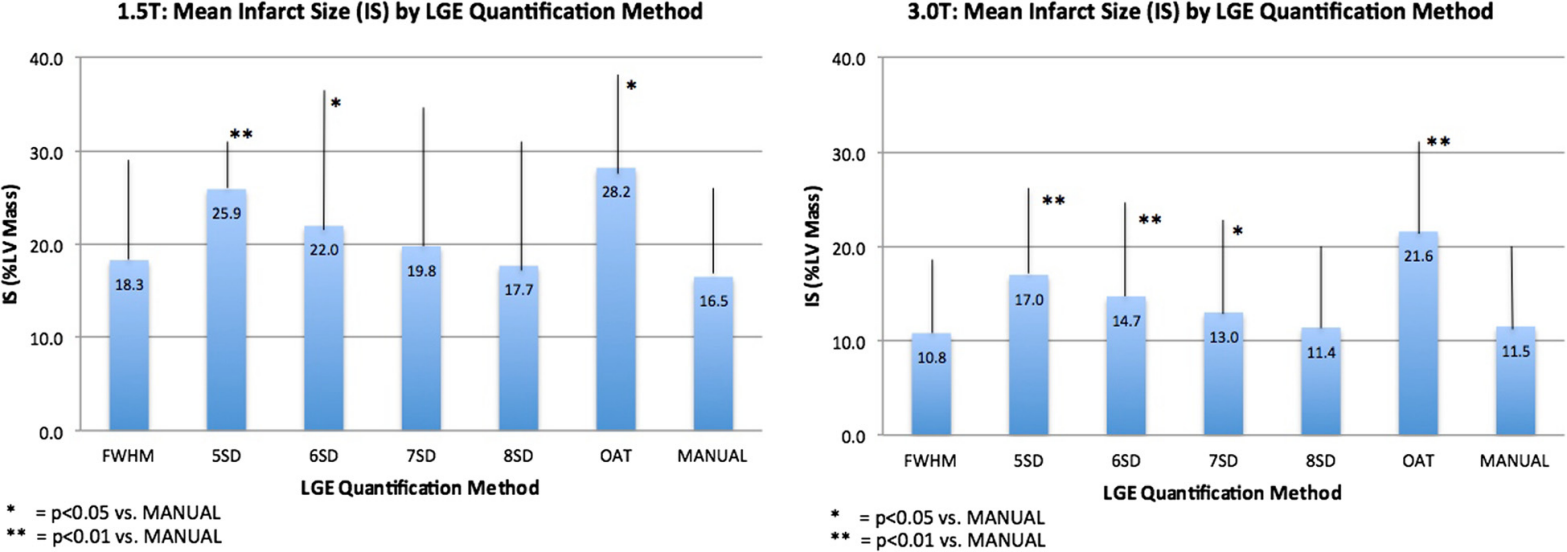
**Mean Infarct Size (IS) by Quantification Method.** *Left panel 1.5T and right panel 3.0T. IS using OAT, 5-8SD, FWHM and manual quantification.* IS = infarct size, FWHM = full-width half maximum, 5-8SD = >5-8 standard deviations, OAT = Otsu’s Automated Thresholding.

**Figure 4:**
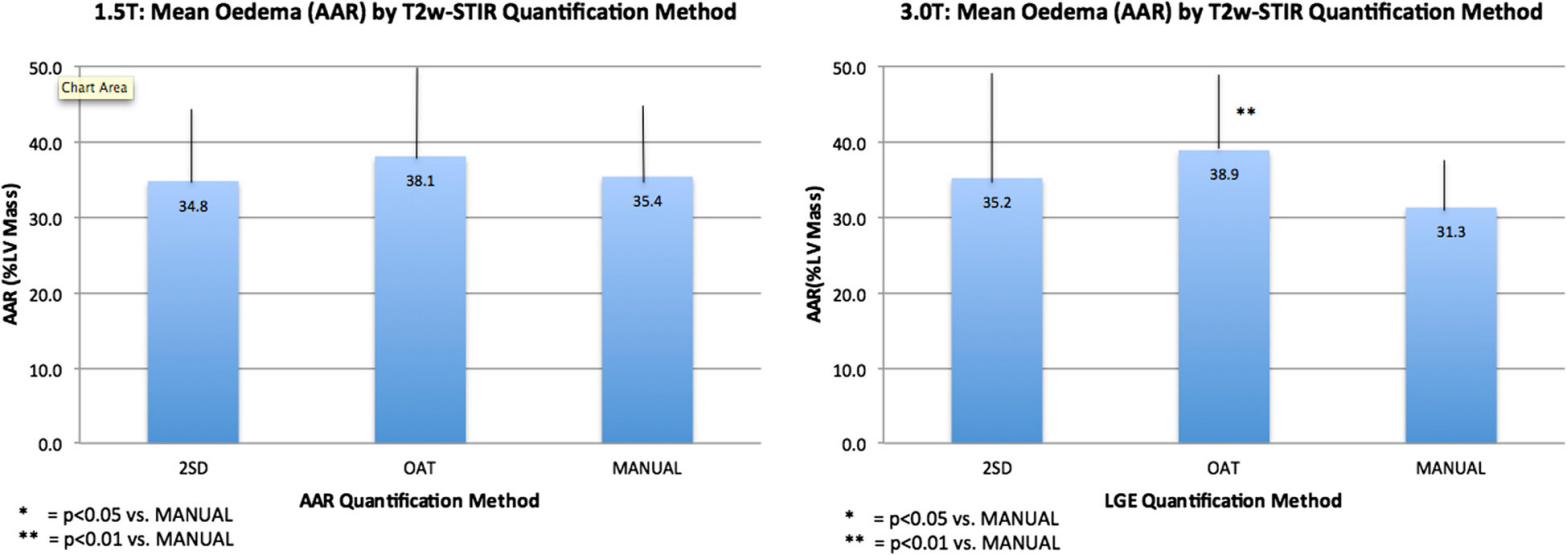
**Mean Area-At-Risk (AAR) by Quantification Method.** *Left panel 1.5T and right panel 3.0T. AAR compared using 2SD, OAT and manual quantification.* IS = infarct size, OAT = Otsu’s Automated Thresholding, 2SD = >2 standard deviations.

Baseline characteristics are summarised in Table 1. Twenty patients were studied (1.5T n = 10, 3.0T n = 10). CMR was undertaken 3.7 ± 1.3 days post STEMI in the 1.5T group and 3.4 ± 2.1 days post STEMI in the 3.0T group. In total, 171 slices were analysed for IS and AAR (89 at 1.5T, at 82 at 3.0T). All LGE and STIR slices were of diagnostic image quality. Data for IS and AAR are shown in Tables 2, 3 and 4, and Figures 3 and 4.

**Table 1:** **Baseline demographics by CMR field strength cohort**

	***1.5T***	***3.0T***
**n**	10	10
**Age (years)**	56.6 ± 14.0	52.6 ± 10.6
**LAD IRA (n, %)**	3 (30%)	4 (40%)
**LCX IRA (n, %)**	2 (20%)	2 (20%)
**RCA IRA (n, %)**	5 (50%)	4 (40%)
**Treatment strategy**	IRA-only PCI: n = 3 (30%) Complete revascularisation: n = 7 (70%)	Vasodilator treatment group: n = 7 (70%) Control group: n = 3 (30%)
**CMR time post STEMI (d)**	3.7 ± 1.3	3.4 ± 2.1
**LVEDM (g)**	111.6 ± 21.9	107.1 ± 23.1
**LVEDV (ml)**	179.9 ± 33.8	169.3 ± 35.2
**LVESV (ml)**	94.7 ± 20.9	94.4 ± 32.3
**LVEF (%)**	47.2 ± 7.8	45.4 ± 8.1
**LGE image quality**	2.5 ± 0.6	2.2 ± 0.6
**T2w-STIR image quality**	2.6 ± 0.5	2.1 ± 0.3

Data expressed as mean ± standard deviation. CMR = cardiovascular magnetic resonance, STEMI = ST-segment elevation myocardial infarction, LVEDM = Left ventricular end-diastolic mass, LVEDV = Left ventricular end-diastolic volume, LVESV = Left-ventricular end-systolic volume, LVEF = Left ventricular ejection fraction, PCI = percutaneous coronary intervention, IRA = infarct-related artery.

**Table 2:** **Infarct size (IS) results at 1.5T by quantification method and corresponding reproducibilities**

***1.5T***	**IS (FWHM)**	**IS (5SD)**	**IS (6SD)**	**IS (7SD)**	**IS (8SD)**	**IS (OAT)**	**IS (MANUAL)**
**Mean IS (%LVM)**	18.3 ± 10.7	25.9 ± 16.1^b^	22.0 ± 15.8^a^	19.8 ± 15.3	17.7 ± 14.4	28.2 ± 11.8^b^	16.5 ± 10.3
**ICC v Manual**	0.909	0.667	0.759	0.804	0.832	0.621	
**Mean bias v Manual (±1.96SD LoA)**	+1.84 (+10.30, −6.62)	+9.39 (+25.58, −6.81)	+5.57 (+21.65, −10.52)	+3.28 (+18.86, −12.30)	+1.21 (+15.92, −13.50)	+11.71 (+17.39, +6.03)	
**Interobserver ICC**	0.922	0.952	0.904	0.906	0.888	0.976	0.793
**Interobserver mean bias (±1.96SD LoA)**	+0.37 (+9.17, −8.43)	+2.54 (+11.62, −6.54)	+4.43 (+16.01, −7.16)	+4.00 (+15.48, −7.47)	+4.01 (+16.02, −8.01)	+0.55 (+5.82, −4.73)	+5.34 (+14.96, −4.28)
**Intraobserver ICC**	0.991	0.957	0.954	0.938	0.925	0.991	0.983
**Intraobserver mean bias (±1.96SD LoA)**	+0.36 (+3.34, −2.61)	−0.81 (+9.37, −10.99)	+0.01 (+10.28, −10.27)	+0.07 (+11.76, −11.62)	+0.42 (+13.00, −12.17)	+0.81 (+4.21, −2.50)	−1.92 (+0.64, −4.49)

IS expressed as mean ± standard deviation. FWHM = full-width half maximum, 5-8SD = >5-8 standard deviation, OAT = Otsu’s Automated Thresholding, LVM = Left Ventricular Mass, ICC = Intraclass Correlation Coefficient, LoA = Limits of Agreement (Bland-Altman).

^a^ - p < 0.05 *vs*. manually quantified IS. ^b^ – p < 0.01 *vs*. manually quantified IS.

**Table 3:** **Infarct size (IS) results at 3.0T by quantification method and corresponding reproducibilities**

***3 T***	**IS (FWHM)**	**IS (5SD)**	**IS (6SD)**	**IS (7SD)**	**IS (8SD)**	**IS (OAT)**	**IS (MANUAL)**
**Mean IS (%LVM)**	10.8 ± 8.2	17.0 ± 11.2^b^	14.77 ± 10.4^b^	13.0 ± 9.7^a^	11.4 ± 9.0	21.6 ± 9.8^b^	11.5 ± 9.0
**ICC v Manual**	0.964	0.780	0.874	0.937	0.966	0.505	
**Mean bias v Manual (±1.96SD LoA)**	+0.22 (+5.09, −4.65)	+6.42 (+14.93, −2.09)	+4.17 (+11.05, −2.71)	+2.38 (+7.92, −3.16)	+0.81 (+5.62, −4.01)	+11.03 (+22.20, −0.15)	
**Interobserver ICC**	0.990	0.957	0.937	0.916	0.888	0.977	0.913
**Interobserver mean bias (±1.96SD LoA)**	−0.49 (+1.74, −2.72)	+0.44 (+7.23, −6.35)	+1.14 (+8.51, −6.23)	+1.40 (+9.25, −6.44)	+1.50 (+9.99, −6.98)	−0.05 (+4.35, −4.44)	+1.97 (+9.48, −5.54)
**Intraobserver ICC**	0.988	0.992	0.992	0.993	0.993	0.986	0.972
**Intraobserver mean bias (±1.96SD LoA)**	+0.20 (+1.49, −1.10)	+0.43 (+2.90, −2.03)	−0.04 (+2.42, −2.50)	+0.10 (+2.19, −1.98)	+0.32 (+2.08, −1.45)	+0.15 (+3.50, −3.21)	+1.14 (+4.35, −2.07)

IS expressed as mean ± standard deviation. FWHM = full-width half maximum, 5-8SD = >5-8 standard deviation, OAT = Otsu’s Automated Thresholding, LVM = Left Ventricular Mass, ICC = Intraclass Correlation Coefficient, LoA = Limits of Agreement (Bland-Altman).

^a^ - p < 0.05 *vs*. manually quantified IS. ^b^ – p < 0.01 *vs*. manually quantified IS.

**Table 4:** **Area at risk (AAR) by field strength and quantification method and corresponding reproducibilities**

		***1.5T***			***3.0T***	
	**AAR (2SD)**	**AAR (OAT)**	**AAR (MANUAL)**	**AAR (2SD)**	**AAR (OAT)**	**AAR (MANUAL)**
**Mean value (AAR [% LVM])**	34.8 ± 9.8	38.1 ± 13.0	35.4 ± 11.2	35.2 ± 14.4	38.9 ± 9.9^a^	30.0 ± 8.2
**ICC v Manual**	0.865	0.920		0.649	0.465	
**Mean bias v Manual (±1.96SD LoA)**	+0.31 (+12.20, −11.57)	+3.62 (+11.24, −4.00)		+5.13 (+22.76, −12.50)	+8.92 (+23.15, −5.31)	
**Interobserver ICC**	0.908	0.976	0.825	0.869	0.981	0.716
**Interobserver mean bias (±1.96SD LoA)**	+3.38 (+9.12, −2.37)	+1.26 (+7.20, −4.68)	+1.31 (+17.11, −14.49)	−0.34 (+15.36, −16.03)	−1.35 (+1.64, −4.33)	−5.20 (+9.54, −19.95)
**Intraobserver ICC**	0.948	0.995	0.977	0.987	0.990	0.826
**Intraobserver mean bias (±1.96SD LoA)**	+2.80 (+7.28, −1.68)	+0.58 (+3.31, −2.16)	−0.01 (+5.62, −5.64)	+1.00 (+5.50, −3.51)	+0.04 (+2.82, −2.74)	+1.46 (+14.84, −11.93)

AAR and MSI expressed as mean ± standard deviation. 2SD = >2 standard deviations, OAT = Otsu’s Automated Thresholding, LVM = Left Ventricular Mass, ICC = Intraclass Correlation Coefficient, LoA = Limits of Agreement (Bland-Altman).

^a^ - p < 0.05 vs. manually quantified AAR.

### Infarct size

**Figure 5:**
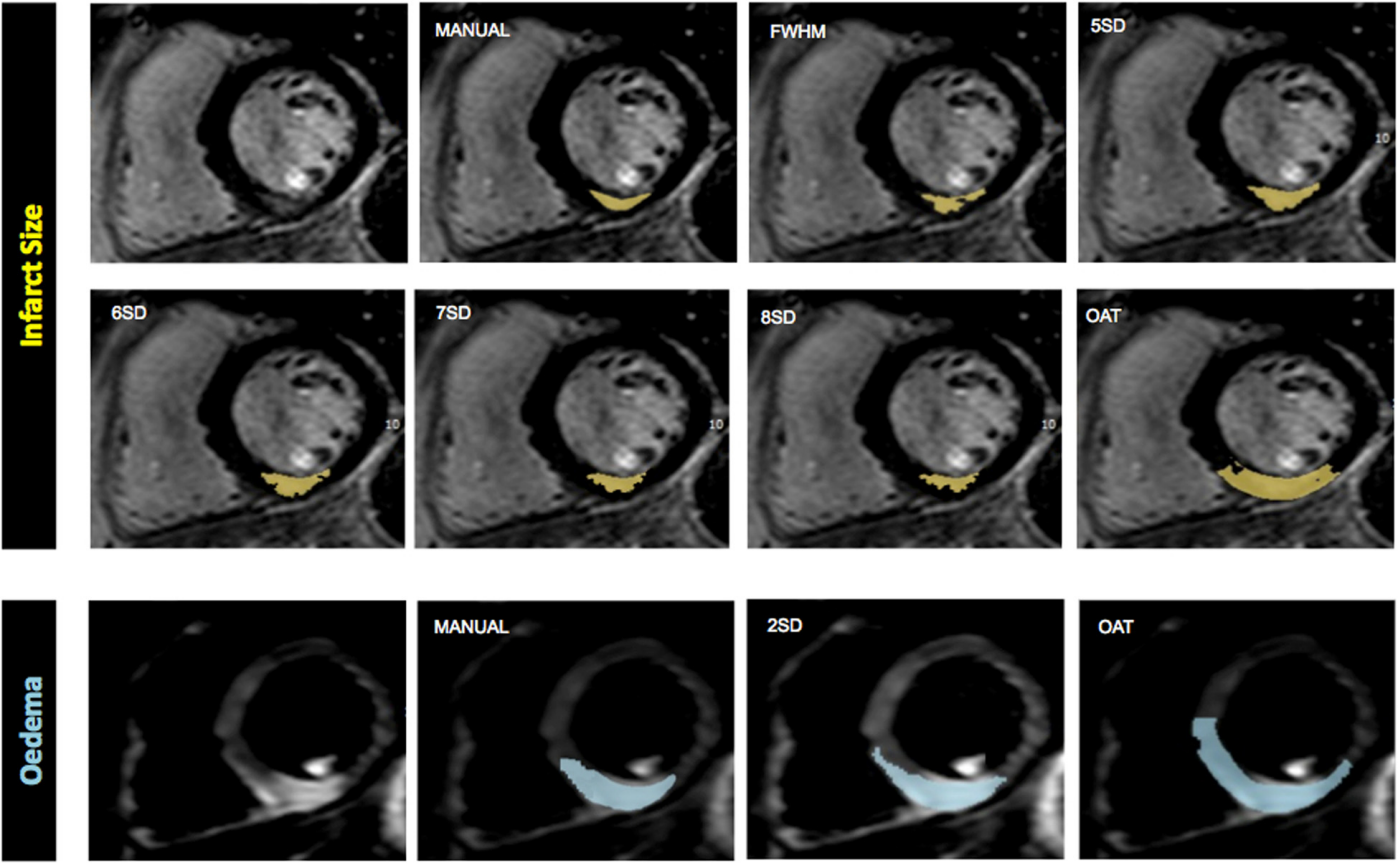
**Infarct size and Area-At-Risk quantification at 3.0T.** *Top/middle rows: IS on a single patient at 3.0T demonstrating increasing IS using FWHM, 5SD and OAT, and decreasing IS from 5–8 SD. FWHM and 8SD closely correlated with the reference standard of manual quantification. Bottom row: AAR in the same patient using OAT and 2SD thresholding was non-significantly greater than the reference standard of manual quantification.* FWHM = full-width half maximum, 2/5/6/7/8SD = >2/5/6/7/8 standard deviations, OAT = Otsu’s Automated Thresholding.

IS varied significantly with the quantification method (Tables 2 and 3 and Figures 3 and 5). FWHM, 7SD and 8SD closely agreed with manual IS quantification at 1.5T, and 6SD showed weak agreement. FWHM and 8SD closely agreed with manual quantification at 3.0T. At both field strengths, IS was significantly greater with OAT and 5SD compared with manual quantification. IS was also greater with 6SD and 7SD at 3.0T. Bland-Altman plots for agreement with manual quantification are shown in Additional file 2. There was a strong trend towards reduced IS quantification time using FWHM compared with all SD-based methods at both field strengths. The reduction in quantification time with FWHM was highly significant when compared with manual quantification at both field strengths, and when compared with 5SD and 8SD at 1.5T. There was no difference in quantification time using FWHM and OAT (Table 5).

**Table 5:** **Time taken per patient for Infarct Size (IS) and Area at risk (AAR) quantification by field strength and quantification method**

	**Time (minutes)**	**p**
.	17.1 ± 2.4 *vs*. 20.9 ± 5.5	0.04
1.5T: FWHM v 6SD (IS)	17.1 ± 2.4 *vs*. 19.4 ± 3.1	0.09
1.5T: FWHM v 7SD (IS)	17.1 ± 2.4 *vs*. 19.1 ± 3.7	0.13
1.5T: FWHM v 8SD (IS)	17.1 ± 2.4 *vs*. 19.6 ± 3.2	<0.01
1.5T: FWHM v OAT (IS)	17.1 ± 2.4 *vs*. 18.0 ± 2.6	0.45
1.5T: FWHM v MANUAL (IS)	17.1 ± 2.4 *vs*. 21.1 ± 4.7	0.01
1.5T: 5SD v OAT (IS)	20.9 ± 5.5 *vs*. 18.0 ± 2.6	0.21
1.5T: 2SD v OAT (AAR)	17.1 ± 2.4 *vs*. 16.7 ± 2.6	0.73
1.5T: 2SD v MANUAL (AAR)	17.1 ± 2.4 *vs*. 18.3 ± 2.6	0.14
1.5T: OAT v MANUAL (AAR)	16.7 ± 2.6 *vs*. 18.3 ± 2.6	0.07
3T: FWHM v 5SD (IS)	18.9 ± 2.7 *vs*. 24.7 ± 9.1	0.08
3T: FWHM v 6SD (IS)	18.9 ± 2.7 *vs*. 22.2 ± 5.2	0.07
3T: FWHM v 7SD (IS)	18.9 ± 2.7 *vs*. 22.5 ± 5.3	0.07
3T: FWHM v 8SD (IS)	18.9 ± 2.7 *vs*. 21.2 ± 3.0	0.08
3T: FWHM v OAT (IS)	18.9 ± 2.7 *vs*. 20.7 ± 3.2	0.11
3T: FWHM v MANUAL (IS)	18.9 ± 2.7 *vs*. 24.0 ± 3.7	<0.01
3T: 5SD v OAT (IS)	24.7 ± 9.1 *vs*. 20.7 ± 3.2	0.25
3T: 2SD v OAT (AAR)	19.6 ± 2.7 *vs*. 18.5 ± 2.6	0.26
3T: 2SD v MANUAL (AAR)	19.6 ± 2.7 *vs*. 18.3 ± 2.6	0.31
3T: OAT v MANUAL (AAR)	18.5 ± 2.6 *vs*. 18.3 ± 2.6	0.83

FWHM = full-width half maximum, 5/6/7/8SD = >5/6/7/8 standard deviation, OAT = Otsu’s Automated Thresholding, 2SD = >2 standard deviations.

p < 0.05 taken as statistically significant.

#### Interobserver and intraobserver variability of IS quantification

Results are displayed in Tables 2 and 3. FWHM and OAT demonstrated extremely high interobserver and intraobserver agreement at both field strengths, with all ICC values >0.922 and mean bias < +1.84%. SD-based techniques demonstrated good interobserver and intraobserver agreement at both field strengths, however lower than for FWHM and OAT, with ICC values >0.888 and mean bias < +4.43%. Interobserver and intraobserver agreement for manual quantification were very high at both field strengths apart from interobserver agreement at 1.5T, which was good (ICC 0.793). Bland-Altman charts for IS are shown in Additional files 2 and 3.

Interobserver agreement for IS at 3.0T was significantly better with FWHM *vs*. manual quantification (p = 0.037). Intraobserver agreement for IS was significantly better at 1.5T with FWHM *vs*. 6SD (p = 0.013), 7SD (p = 0.022) and 8SD (p = 0.037), and at 3.0T for FWHM *vs*. manual (p = 0.047). There was a strong trend towards higher intraobserver agreement for IS at 1.5T with FWHM vs. manual (p = 0.093).

#### Correlation of myocardial injury with LV ejection fraction

At 1.5T, FWHM and manual quantification demonstrated a strong inverse correlation between IS and LVEF (FWHM: r = −0.745, p = 0.013; manual r = −0.709, p = 0.022). All other methods demonstrated moderate inverse correlation and did not reach statistical significance. At 3.0T, FWHM IS showed a significant, moderate correlation with LVEF (r = −0.673, p = 0.033). The correlation using all other techniques was weaker and not statistically significant.

### AAR extent

AAR varied with the quantification method used (Figures 4 and 5). There was no significant difference between 2SD, OAT and manually quantified AAR at 1.5T. At 3.0T, AAR quantified with OAT was larger than that manually contoured (p = 0.004) and similar to that on 2SD. Agreement with manual quantification at 1.5T tended to be higher for OAT than 2SD, with ICC 0.920 and narrower limits of agreement on Bland-Altman analysis. There was no difference in AAR quantification time using OAT, 2SD or manual quantification at 1.5T or 3.0T (Table 5), Additional files 4 and 5.

#### Interobserver and intraobserver variability of AAR and MSI quantification

OAT had extremely high interobserver and intraobserver agreement for AAR quantification at both field strengths, with all ICC values >0.976. Good interobserver agreement was seen for 2SD quantification of AAR at both field strengths. Manual quantification demonstrated excellent interobserver agreement at 3.0T. Interobserver agreement at 1.5T and intraobserver agreement at both field strengths was good with manual quantification (ICC >0.716).

Interobserver agreement at 3.0T was significantly better for OAT vs. manual quantification (p = 0.017), and at 1.5T was borderline significantly higher for OAT vs. manual (p = 0.059). Intraobserver agreement at 3.0T was significantly better for OAT vs. manual quantification (p = 0.007). The raw datasets for IS and AAR quantification at 1.5T and 3.0T field strengths are available in Additional files 6 and 7.

## Discussion

In this study we assessed IS and AAR quantification in acute STEMI patients with currently used semi-automatic techniques at 1.5T and 3.0T. FWHM and 8SD closely agreed with the reference standard of manual IS quantification at both field strengths, whereas 5SD and OAT led to higher IS values compared to manual quantification. AAR measured by OAT and 2SD were similar to manual quantification only at 1.5T. Interobserver and intraobserver agreement for IS and AAR quantification were better with FWHM and OAT compared with manual quantification respectively, and tended to be better than with SD-based methods. There was an inverse correlation between IS and LVEF for all quantification methods and this was strongest and most significant for FWHM. Our study is the first to assess IS quantification methods using 7SD and 8SD thresholding and to assess IS and AAR quantification at 3.0T.

### Mean IS using the quantification techniques

LGE IS quantification in acute MI has been validated in a small number of animal studies. FWHM [8] and manual quantification [9] of *in-vivo* images closely correlated with IS on tetrazolium chloride stained canine hearts. Kim et al. [7] demonstrated good agreement of 2SD thresholding with tetrazolium chloride stained canine myocardium. However this was on *ex-vivo* slices with high spatial resolution and in the absence of rhythm and motion artefacts, and may not be generalizable to humans [7]. Indeed, 2SD has been shown to overestimate IS in humans based on functional improvement and IS reduction in enhanced areas.[26,27] There is no histological validation in humans and hence no ‘gold standard’ quantification. We thus used manual assessment as has been used previously [6,12], however derived from the mean of repeated analyses by three experienced CMR cardiologists to increase the robustness of our reference standard.

FWHM and 8SD were the only methods in our study showing good agreement with manual quantification at both field strengths. This may be because they are less prone to IS overestimation resulting from oedema and partial volume effects giving rise to intermediate signal intensities [26,28]. This resulted in negligible requirements for manual exclusion of noise artefact with FWHM and 8SD. This in conjunction with the relative ease in identification of the brightest infarct core compared with deciding on a representative remote ROI is likely to explain the shorter time required for IS quantification using FWHM compared with SD-based techniques.

The greater IS using 5SD compared with manual quantification in our study is in agreement with previous results at 1.5T [6]. These findings indicate that the good agreement between 5SD and manual quantification in chronic ischaemic heart disease [29], where infarct tends to have a higher and more homogenous SI [6], cannot be extrapolated to acute STEMI patients. The close correlation of 5SD and in particular OAT with manual assessment shown by Vermes et al. [12] is in contrast to our findings. IS quantification was only performed on slices with infarct seen visually in that study, thus potentially underestimating IS. In addition, the small remote ROI used for 5SD thresholding (0.5-1 cm^2^) by Vermes et al. may not adequately represent remote myocardium signal intensity, thus leading to underestimation or overestimation of IS if an excessively bright or dark, isolated region of myocardium is taken as the remote ROI respectively. By setting the ROI size at 2 cm^2^ for all SD-based methods in our study, we aimed to ensure that the ROI was large enough to represent remote myocardium accurately. Using the same remote ROI for all SD-based methods in our study ensured consistency and removed the effect of ROI size and location when comparing IS between 5–8 SD thresholds. Hence, 6 and 5-SD and 7, 6 and 5-SD quantification overestimated IS at 1.5T and 3.0T respectively due to their intrinsically progressively lower signal intensity thresholds and not due to differences in remote ROI.

**Figure 6:**
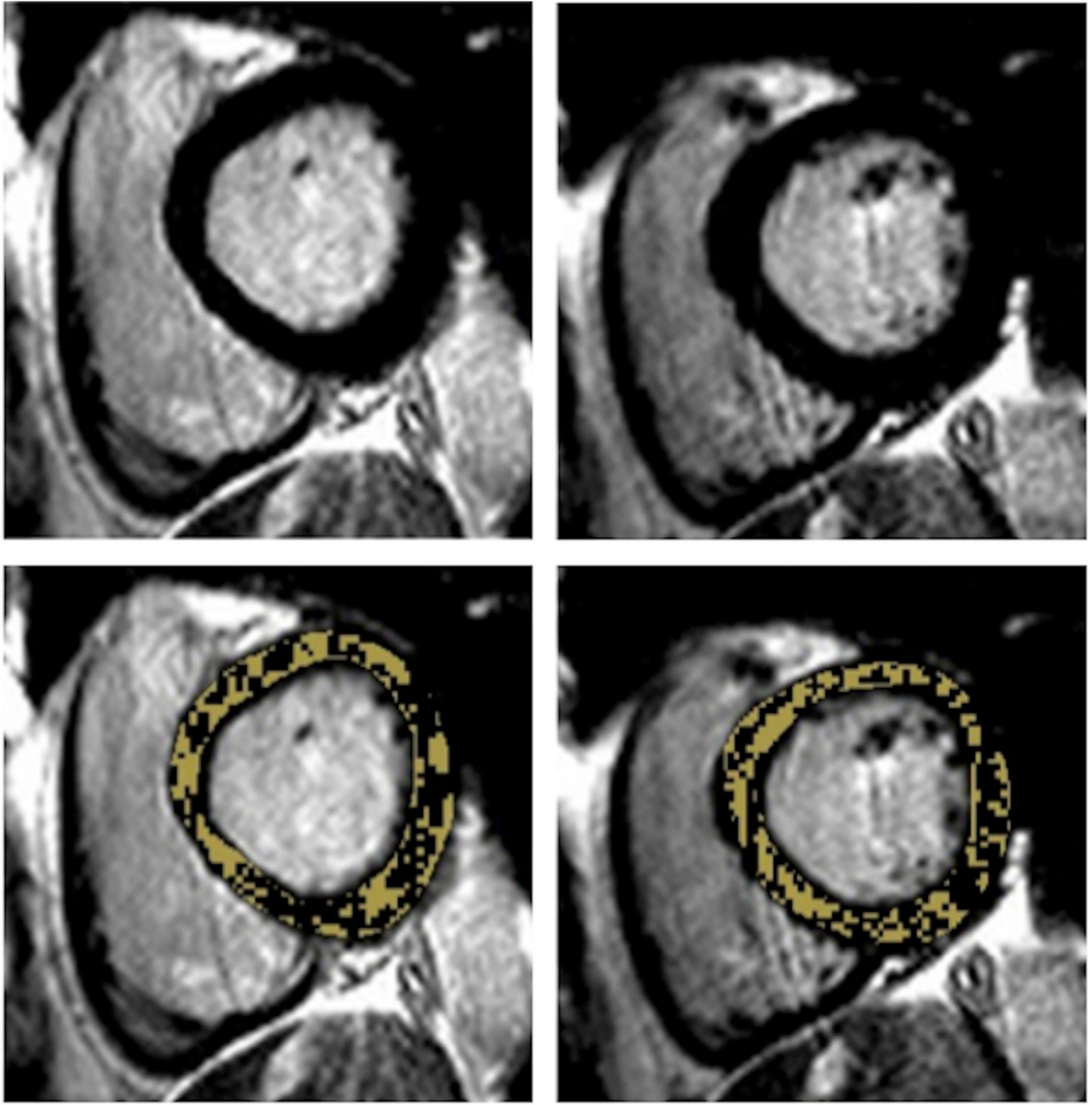
**Hyperenhancement with OAT without obvious Infarct.** *In this case there is no infarct present (top row), whereas OAT has calculated a significant infarct volume (bottom row).* OAT = Otsu’s Automated Thresholding.

OAT has the potential to overestimate LGE IS because it calculates an individual SI threshold, and thus enhancement on *every* slice, regardless of the presence of infarct (Figure 6). Whilst small areas of enhancement in the non-infarct region were manually excluded, it is likely that OAT leads to higher values due to near transmural enhancement in the infarct area, in the presence of peri-infarct oedema [11].

We studied IS and AAR quantification early after STEMI. IS decreases with time post PPCI with a reduction of ~30% demonstrated within the first week in some studies [26,30]. The extent of necrosis is overestimated by LGE early post STEMI due to cellular disruption and oedema. As scar resorbs and remodels, IS reduces and scar may become more homogenous in signal intensity and brighter. The relative overestimation of IS by lower standard deviation thresholds and OAT compared with FWHM, 8SD and manual quantification may thus be more significant in acute compared with in chronic infarcts. We chose an early time point to minimise drop-out in the study and most importantly, all the data relating infarct size to subsequent prognosis following STEMI has been based on early measurement of infarct size (usually within 1 week) [4,31]. Whether AAR varies in the first week after STEMI has shown conflicting results [26,27]. As we have only scanned the patients in this study on a single occasion we cannot comment on how the results would have varied if performed at later dates following presentation.

### Interobserver and intraobserver variability of IS quantification

The excellent interobserver and intraobserver agreement for FWHM, 5SD and OAT quantification of IS in our study at both field strengths is in agreement with previous studies at 1.5T: FWHM, 5SD [6,27] and FWHM, OAT [12]. Consistent with the work of Flett et al. [6], we found that the FWHM technique had greater interobserver and intraobserver reproducibility compared with SD-based and manual quantification. This is expected when considering that for each patient there is a single brightest core of infarct. This is in contrast to the remote ROI, which could be drawn on any slice without complete LGE in SD-based quantification, or manual contouring of enhancement, which is completely user-dependent and in the acute phase post STEMI could potentially be affected by partial volume in infarct boundaries and oedematous myocardium.

### Mean AAR and MSI using the quantification techniques

T2w-STIR AAR is typically quantified using 2SD thresholding. Validation studies are limited. 2SD-derived AAR on T2w images *in vivo* correlated with microsphere-assessed AAR in canine myocardium (r = 0.84).[32] There is no gold standard AAR quantification method on T2w-STIR, hence we used manual assessment.

The close agreement between OAT and manually contoured AAR at 1.5T is consistent with the work of McAlindon et al.[14] OAT however demonstrated greater AAR compared with manual quantification at 3.0T. This is in keeping with Sjogren et al. [13] who showed overestimation of AAR using OAT with a mean bias of +5.3 ± 9.6% compared with manual quantification [13]. The determination of an optimal threshold and quantification of enhancement on every slice with OAT, regardless of oedema is likely to contribute to this. The risk of overestimation of AAR will be greatest in slices with minimal oedema since OAT will deem a proportion of pixels enhanced. This may potentially have contributed to the overestimation of AAR at 3.0T in our study with OAT, since our 3.0T cohort had a smaller AAR than the 1.5T patients. IS was also smaller in our 3.0T cohort and may have contributed to the greater overestimation of IS using OAT at 3.0T compared with 1.5T. Conversely, underestimation of AAR is more likely in slices with complete enhancement since OAT will deem a proportion of pixels unenhanced [13]. T2w-STIR images were of diagnostic quality in all patients in our study, however mean quality control grading was slightly lower at 3.0T (2.1 ± 0.3 [3 T] vs. 2.6 ± 0.5 [1.5T], p = 0.05) and may have potentially contributed to the overestimation of OAT-derived AAR if there was more noise artefact in the AAR or signal intensity drop out in remote regions by reducing the threshold. More work into automated quantification methods is required, in particular at 3.0T. Newer automated techniques, taking into account *a priori* information about the culprit artery [13] and including noise and false positive artefact exclusion [10,21] algorithms may improve the accuracy of automated IS and AAR quantification.

The relative degree of AAR overestimation in our study was, however, considerably less than for IS. The predominantly transmural pattern of OAT enhancement for IS and AAR may cause less overestimation of AAR compared with IS, since oedema has been shown to be predominantly transmural in 70-100% of oedematous segments [33,34].

### Interobserver and intraobserver variability of AAR quantification

The relatively low interobserver and intraobserver agreement using 2SD compared with OAT at both field strengths is likely to result from varying manual definition of the remote ROI. The extremely high ICC’s obtained with OAT are remarkable considering that these figures still take account differences in manual correction and contouring of endocardial and epicardial borders. Given these results, quantification of AAR with OAT could minimise variability in measurement in multi-centre trials.

### Limitations

The main limitation of our and previous similar studies is the lack of a gold standard for IS and AAR quantification. Different quantification techniques were studied for IS and AAR. FWHM quantification of AAR was not undertaken due to the lower CNR of T2w-STIR imaging, since the vast majority of myocardium would have signal intensity >50% of the maximum at the AAR core, leading to potentially extreme overestimation of AAR and MSI. Indeed, McAlindon et al. demonstrated that FWHM significantly overestimated AAR compared to all other quantification methods tested at 1.5T (2,3,5 SD, OAT, manual quantification) [14]. 5SD thresholding was not assessed for AAR as it has never been validated or correlated with clinical outcomes and the only study to feature it demonstrated that it significantly overestimated AAR compared to all other quantification methods tested at 1.5T (2,3 SD, FWHM, OAT, manual quantification) [14]. 2SD thresholding was not assessed for IS as it has been shown to overestimate IS [6,12] and had the lowest correlation with histological IS on tetrazolium chloride staining using Bland-Altman analysis [8] compared with all other quantification methods used in studies of IS in acute STEMI (5SD, FWHM, manual quantification). Test-retest reproducibility was not assessed and should be considered in future studies. Infarct heterogeneity and identification of peri-infarct zone (greyzone) was not assessed in this study and may be of interest to assess in future studies using OAT. We deliberately studied patients imaged at different field strengths and with different scanner vendors to represent the situation in multi-centre clinical trials and this should make the results more generalizable. Our sample size (total n = 20) is limited, however is comparable to similar studies in myocardial infarction [8-10,12,14,20] and our findings are supported by their consistency at both field strengths.. Finally our results may not be generalizable to if patients are scanned at different time points following STEMI.

## Conclusions

Inter- and intraobserver variability for the quantification of IS with FWHM is excellent at 1.5 and 3.0T and better than when using manual quantification. Only FWHM and 8SD closely agreed with manual delineation of IS at both field strengths. FWHM had better reproducibility, shorter quantification time and closer correlation with LVEF and may be the preferred method for IS quantification in future studies. AAR is similar when assessed with OAT, 2SD and manual quantification at 1.5T, however OAT has excellent intra and interobserver variability and thus has potential in quantification of AAR at 1.5T, especially in multi-centre studies. OAT overestimated AAR at 3.0T compared with manual quantification and thus cannot currently be recommended as the preferred method for AAR quantification at 3.0T.

## Additional files

Additional file 1:
**CMR parameters for T2-weighted STIR and late gadolinium enhanced (LGE) sequences on the scanners used.**
Additional file 2:
**Infarct Size (IS) Quantification at 1.5T using the quantification techniques.**
Additional file 3:
**Infarct Size (IS) Quantification at 3.0T using the quantification techniques.**
Additional file 4:
**Area At Risk (AAR) Quantification at 1.5T using the quantification techniques.**
Additional file 5:
**Area At Risk (AAR) Quantification at 3.0T using the quantification techniques.**
Additional file 6:
**1.5T dataset for IS and AAR quantification.**
Additional file 7:
**3.0T dataset for IS and AAR quantification.**
