# Supplementary material for: Comparison of semi-automated methods to quantify infarct size and area at risk by cardiovascular magnetic resonance imaging at 1.5T and 3.0T field strengths
Source: BMC Res Notes. 2015 Feb 25;8:52. doi: 10.1186/s13104-015-1007-1 (PMC4347654; doi:10.1186/s13104-015-1007-1)
Supplement: Additional file 1: — CMR parameters for T2-weighted STIR and late gadolinium enhanced (LGE) sequences on the scanners used. [file 13104_2015_1007_MOESM1_ESM.docx]

**Supplemental Data 1: CMR parameters for T2-weighted STIR and late gadolinium enhanced (LGE) sequences on the scanners used**

| **Scan platform** | **T2w-STIR parameters (area at risk)** | **LGE parameters (infarct size)** |
| --- | --- | --- |
| Siemens Avanto 1.5T | 10mm slice thickness, no gap, matrix 208-256x256, FOV ~300-360x360-420, echo train length 20-40, coil signal intensity correction on, TR 2.1s, TE 47ms, flip angle 180^o^ | 8mm slice thickness, 2mm gap, matrix 208x256, FOV 300x400, TI 220-360ms (progressive optimization), TR 700ms, TE 4.9ms, flip angle 30^o^ |
| Philips Intera 1.5T | 10mm slice thickness, no gap, matrix 208-256x256, FOV ~300-360x360-420, echo train length (20-40), coil signal intensity correction on, TR 2.2s, TE 60ms, flip angle 90^o^ | 8mm slice thickness, 2mm gap, matrix 208-256x256, FOV 300x400, TI 220-360ms (progressive optimization), TR 4.5ms, TE 1.8ms, flip angle 15^o^ |
| Siemens Skyra 3.0T | 10mm slice thickness, no gap, matrix 208-256x256, FOV ~300-360x360-420, echo train length (20-40), coil signal intensity correction on, TR 1.9s, TE 44ms, flip angle 180^o^ | 10mm slice thickness, no gap, matrix 208-256x256, FOV 300x400, TI 220-360ms (progressive optimization), TR 900ms, TE 2.0ms, flip angle 20^o^ |
| Philips Acheiva 3.0T | 10mm slice thickness, no gap, matrix 208-256x256, FOV ~300-360x360-420, echo train length (20-40), coil signal intensity correction on, TR 2.2s, TE 60ms, flip angle 90^o^ | 10mm slice thickness, no gap, matrix 208-256x256, FOV 300x400, TI 220-360ms (progressive optimization), TR 6.1ms, TE 3.0ms, flip angle 25^o^ |
| GE Signa HDxt 3.0T | 10mm slice thickness, no gap, matrix 208-256x256, FOV ~300-360x360-420, echo train length 20-40, coil signal intensity correction on, TR 2.5s, TE 62ms, flip angle 90^o^ | 10mm slice thickness, no gap, matrix 208-256x256, FOV 300x400, TI 220-360ms (progressive optimization), TR 6.5ms, TE 3.1ms, flip angle 20^o^ |
