# Supplementary material for: Comparison of semi-automated methods to quantify infarct size and area at risk by cardiovascular magnetic resonance imaging at 1.5T and 3.0T field strengths
Source: BMC Res Notes. 2015 Feb 25;8:52. doi: 10.1186/s13104-015-1007-1 (PMC4347654; doi:10.1186/s13104-015-1007-1)
Supplement: Additional file 2: — Infarct Size (IS) Quantification at 1.5T using the quantification techniques. [file 13104_2015_1007_MOESM2_ESM.docx]

**Supplemental Data 2 – Infarct Size (IS) Quantification at 1.5T using the quantification techniques**

**Supplemental Data 2 – Infarct Size (IS) Quantification at 1.5T using the quantification techniques**

**Supplemental Data 2 – Infarct Size (IS) Quantification at 1.5T using the quantification techniques**

**Supplemental Data 2 – Infarct Size (IS) Quantification at 1.5T using the quantification techniques**
